# Nutritional status and physical activity levels of adolescents in public and private schools

**DOI:** 10.15649/cuidarte.3942

**Published:** 2024-11-06

**Authors:** María V Arias-Silva, Valentina Pedreros-Lemuz, Angie L Rodríguez-Perdomo, Raúl A González-Lozano, Jorge A Ramos-Castaneda

**Affiliations:** 1 Faculty of Nursing, Universidad Antonio Nariño, Neiva, Colombia. E-mail: marias10@uan.edu.co Universidad Antonio Nariño Faculty of Nursing Universidad Antonio Nariño Neiva Colombia marias10@uan.edu.co; 2 Faculty of Nursing, Universidad Antonio Nariño, Neiva, Colombia. E-mail: vpedreros66@uan.edu.co Universidad Antonio Nariño Faculty of Nursing Universidad Antonio Nariño Neiva Colombia vpedreros66@uan.edu.co; 3 Faculty of Nursing, Universidad Antonio Nariño, Neiva, Colombia. E-mail: angierodriguez56@uan.edu.co Universidad Antonio Nariño Faculty of Nursing Universidad Antonio Nariño Neiva Colombia angierodriguez56@uan.edu.co; 4 Research Group Innovación y Cuidado, Faculty of Nursing, Universidad Antonio Nariño, Bogotá, Colombia. E-mail: ragonzalez75@uan.edu.co Universidad Antonio Nariño Research Group Innovación y Cuidado Faculty of Nursing Universidad Antonio Nariño Bogotá Colombia ragonzalez75@uan.edu.co; 5 Research Group Innovación y Cuidado, Faculty of Nursing, Universidad Antonio Nariño, Neiva, Colombia. E-mail: joramos98@uan.edu.co Universidad Antonio Nariño Research Group Innovación y Cuidado Faculty of Nursing Universidad Antonio Nariño Neiva Colombia joramos98@uan.edu.co

**Keywords:** Exercise, Sedentary Behavior, Adolescent, Obesity, Nutritional Status, Ejercicio Físico, Conducta Sedentaria, Adolescente, Obesidad, Estado Nutricional, Exercício Físico, Comportamento Sedentário, Adolescente, Obesidade, Condição Nutricional

## Abstract

**Introduction:**

Obesity and physical inactivity in adolescents are associated with some diseases, and poor academic performance. Studies have reported differences in the level of physical activity according to the type of school.

**Objective:**

To compare the prevalence of obesity and physical inactivity in adolescents in a public and a private school.

**Materials and Methods:**

A cross sectional study was conducted among adolescents attending a public school and a private school. Ninth, tenth, and eleventh grade students between 14 and 17 years were included. Physical activity level was assessed using the Physical Activity Questionnaire for Adolescents, and nutritional status using body mass index for age.

**Results:**

Information was collected from 343 students, of whom 55.10% (n=189) were from a public school. The 28.28% were overweight. The overall level of physical activity in adolescents was low (mean= 1.97 95% CI 1.92 - 2.01). It was found that public school students were more inactive adjusted (PR= 1.40; 95% CI 1.03 - 1.92) compared to private school students.

**Discussion:**

Adolescents do not achieve optimal levels of frequency and duration of physical activity, highlighting the need for educational programs and attractive interventions to promote physical activity.

**Conclusion:**

Adolescent students have low levels of physical activity, with more inactive students in the public school compared to the private school. The prevalence of overweight was high in both schools.

## Resumen.

**Introducción:**

La obesidad y la inactividad física en adolescentes se asocia con múltiples enfermedades y bajo rendimiento escolar. Algunos estudios han reportado diferencias en nivel de actividad física, práctica deportiva o de clases de educación física según la naturaleza de la institución educativa.

**Objetivo:**

Comparar la prevalencia de obesidad e inactividad física en adolescentes de una institución educativa pública y una privada.

**Materiales y Métodos:**

Se realizó un estudio de corte transversal en adolescentes escolarizados en una institución educativa pública y una privada. Se incluyeron estudiantes de grado noveno, décimo y once de edades comprendidas entre los 14 y 17. Se evaluó el nivel de actividad física mediante el instrumento Physical Activity Questionnaire para adolescentes, y el estado nutricional mediante el índice de masa corporal para la edad.

**Resultados:**

Se recolectó información de 343 estudiantes, donde el 55,10% (n= 189) fueron estudiantes de una institución educativa pública. El 28,28% presentaron exceso de peso. El nivel global de actividad física en los adolescentes fue bajo (media= 1,97 IC95% 1,92 - 2,01). Se identificó que los estudiantes de la institución educativa pública son más inactivos (RP = 1,40; IC95% 1,03 - 1,92) comparado con los de la institución privada.

**Discusión:**

Los adolescentes no alcanzan niveles óptimos de frecuencia y duración de la actividad física, por lo que se demuestra la necesidad de diseñar programas educativos e intervenciones atractivas para promover la actividad física.

**Conclusión:**

Los estudiantes adolescentes presentan un bajo nivel de actividad física, siendo más inactivos los estudiantes de la institución educativa pública, en comparación con la privada. La prevalencia de exceso de peso fue alta en ambas instituciones educativas.

## Resumo.

**Introdução:**

A obesidade e a inatividade física em adolescentes estão associadas a múltiplas doenças e ao mau desempenho escolar. Alguns estudos relataram diferenças no nível de atividade física, prática esportiva ou aulas de educação física dependendo da natureza da instituição de ensino.

**Objetivo:**

Comparar a prevalência de obesidade e inatividade física em adolescentes de uma instituição de ensino pública e privada.

**Materiais e Métodos:**

Foi realizado um estudo transversal entre adolescentes matriculados em uma instituição de ensino pública e privada. Foram incluídos alunos do nono, décimo e décimo primeiro ano entre 14 e 17 anos. O nível de atividade física foi avaliado pelo Questionário de Atividade Física para Adolescentes e o estado nutricional pelo índice de massa corporal para idade.

**Resultados:**

Foram coletadas informações de 343 alunos, sendo 55,10% (n= 189) alunos de uma instituição pública de ensino. 28,28% estavam acima do peso. O nível global de atividade física dos adolescentes foi baixo (média = 1,97 IC 95% 1,92 - 2,01). Identificou-se que os alunos da instituição de ensino pública são mais inativos fisicamente (RP = 1,40; IC95% 1,03 - 1,92) em comparação aos da privada.

**Discussão:**

Os adolescentes não atingem níveis ótimos de frequência e duração da atividade física, evidenciando a necessidade de programas educacionais e intervenções atrativas para promover a atividade física.

**Conclusão:**

Os estudantes adolescentes apresentam baixo nível de atividade física, sendo os estudantes de instituições de ensino públicas mais inativos em comparação aos privados. A prevalência de excesso de peso foi elevada em ambas as instituições de ensino.

## Introduction

Obesity is a chronic, multifactorial disease that is considered a public health problem due to its high prevalence worldwide and its association with chronic diseases^1^. Obesity is considered a risk factor for the development of cardiovascular diseases, stroke, osteoarticular pathologies, diabetes mellitus, and some cancers (endometrial, breast, ovarian, liver, colon, and others) ^2^^, ^^6^.

It is estimated that 20% of children and adolescents worldwide are overweight or obese, with some countries such as the United States, Mexico, Canada, Chile, and Argentina having the highest prevalence^7^. Latin America and the Caribbean have one of the highest prevalence of obesity in the world among people aged 5 to 19^8^. In addition, it has been determined that more than 5 million people die each year as a result of being overweight or obese^9^. In Colombia, according to the National Survey ofthe Nutritional Situation ofColombia (ENSIN, for its acronym in Spanish), obesity has increased among children and adolescents compared to previous years^10^.

A major cause of obesity is the energy imbalance between calories consumed and expended, with diet being an important factor^11^. Consumption of foods high in saturated fat, salt, and sugar, or ultra-processed foods significantly contributes to obesity^11^. Another relevant aspect that causes obesity is exogenous determinants such as a sedentary lifestyle and physical inactivity (PI) ^12^.

According to the World Health Organization, people who are insufficiently physically active (PA) have a 30% higher risk of death than those who engage in moderate physical activity^13^. In addition, several studies have shown that youth who do not engage in PA are at greater risk for mental health problems, poor academic performance, reduced quality of life, and the development of chronic noncommunicable diseases^14^^, ^^18^. A recent systematic review found that physically inactive students are at higher risk of anxiety, obesity, depression, and poor diet^19^. One of the main factors identified for physical inactivity is excessive smartphone use, which leads to greater perceived stress and poor sleep quality in adolescents^20^.

In Latin America and the Caribbean, about 84% of adolescents do not get enough PA, while in high-income countries, the prevalence drops to 78%^21^. Some studies have reported differences in PA levels, sports practice, physical education classes, or sedentary behaviors among school-aged adolescents according to social determinants, such as economic income, mother education^22^, or type of school (Private or Public)^23^. However, a recent study among adolescents in the United States found no difference in PA levels when comparing private and public schools^24^. Therefore, there is not yet sufficient evidence to demonstrate a difference in the level of PA according to the type of school. This study aimed to compare the prevalence of obesity and physical inactivity (PI) in adolescents aged 14 to 17 years in a public and a private school.

## Materials and Methods

### Study design

A cross-sectional study with an analytical approach was conducted on adolescents attending a public school and a private school. The public school is located in the municipality of Campoalegre (Huila) and has approximately 285 students in secondary grades. The private school is in the municipality of Neiva (Huila), with approximately 210 secondary school students. The data collection process was conducted between May and July 2023. 

### Participants

The study included ninth, tenth, and eleventh graders between the ages of 14 and 17 enrolled in Campoalegre’s public and Neiva’s private schools for the year 2023. Students with reduced mobility were excluded because the instrument was designed for people without mobility limitations.

### Sample size

Considering that the population consisted of 353 students in the ninth, tenth, and eleventh grades in both schools, it was decided to conduct a consecutive sampling^25^, including all students who met the inclusion criteria, had parental consent, and gave their assent during recruitment period.

### Variables

The outcome variable was the level of PA assessed by the PAQ-A. A categorization was made to determine PI, considering the median PA. The students who scored < 1.90 were considered inactive. The independent variables were sex, age, grade level, type of school (private or public), socioeconomic stratification, and anthropometric status. Dataset can be download in Mendeley Data^26^.

### Data source and measurement

A properly calibrated Prestige digital scale and a tape measure were used for anthropometric assessment. Body mass index (BMI)-for-age and height-for-age (H/A) were used to determine the cutoff value for each student according to sex, taking into account the cutoff values of Ministry of Health and Social Protection of Colombia^27^.

PA was assessed using the Physical Activity Questionnaire for Adolescents (PAQ-A), which assesses adolescents’ activity over the past seven days in various areas of daily life^28^. The questionnaire consists of 9 closed-ended quantitative questions, each item rated on a Likert scale, commonly used to measure opinions (44). The PAQ A was developed in Spain, demonstrating good reliability with an intraclass correlation coefficient of 0.71, and was shown to have a moderate correlation (rho = 0,39, P <0,001) to classify physical activity levels with respect to the accelerometer^29^. Some studies already use this questionnaire in Colombia^30^^, ^^31^.

### Bias

In order to avoid selection bias, it was decided to use consecutive sampling to include all adolescents who chose to participate in the study; this ensured that the data would be representative of the population of the two educational institutions. To avoid measurement bias, researchers were trained in the use of the PAQ-A, an instrument with validity and reliability used in multiple studies with a similar population^29^.

### Statistical methods

Categorical variables were analyzed using absolute and relative frequencies, while quantitative variables were analyzed using means and standard deviations. The variables of age, sex, and socioeconomic stratification were compared by type of school (public or private). The prevalence of nutritional alteration was then determined, considering the BMI-for-age and height-for-age, as well as by the type of school. Later, the global level of PA was calculated by taking the average score of the eight questions of the PAQ-A. Comparison of each questionnaire item by type of school was made using the prevalence ratio (PR) with 95% confidence intervals. In order to identify factors associated with PI, the global score was categorized by the median value as inactive for those students scoring < 1.90 and physically active for those scoring > 1.90. This result was compared by sex, type of school, grade level, socioeconomic stratification, and excess weight (overweight or obesity). A log-binomial regression model was built for explanatory purposes, including type of school as an independent variable and adjusting for covariates such as sex, grade level, and excess weight.

### Ethical considerations

The study was submitted to and approved by the Universidad Antonio Nariño research ethics committee (record 44-2023) and participating institutions. The project was socialized to administrators, teachers, students, and parents of each school. The informed consent was completed by the parents of the participants, and each adolescent completed the informed assent.

## Results

**Figure 1:**
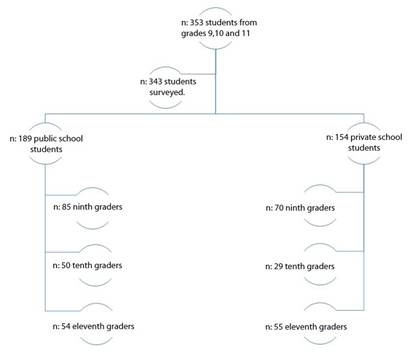
Study Sample Selection Flowchart.

Data were collected from 343 students who volunteered to participate in the study. 55.10% (n=189) were students from the public school, and 44.90% (n=154) from the private school. The 45.20% of students were 9th graders (Figure 1).

The mean age of the students surveyed was 15.6 (SD=0.727) years, and 52.48% were female. It was observed that 82.80% of the students were from socioeconomic strata 1 and 2, and there were no students from socioeconomic strata 3 in the public school (Table 1).

**Table 1:** Distribution by age, socioeconomic stratification, and sex of the students surveyed.

	Public institution n (%)	Private institution n (%)	Total n (%)
Age (years)			
Mean (SD)	15.7 (0.771)	15.6 (0.665)	15.6 (0.727)
Sex			
Female	89 (47.09)	91 (59.09)	180 (52.48)
Male	100 (52.91)	63 (40.91)	163 (47.52)
Stratification			
I	113 (59.79)	13 (8.44)	126 (36.73)
II	76 (40.21)	82 (53.25)	158 (46.06)
III	0 (0	59 (38.31)	59 (17.20)
Total	189	154	343

*SD: Standard deviation*

**Figure 2:**
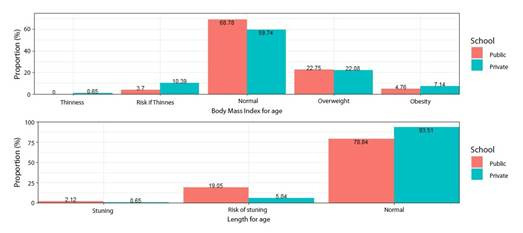
Nutritional status, body mass index for age, and height for age.

The prevalence of nutritional alteration was 35.78%, slightly higher among adolescents in the private school (40.26%) compared to the public school (31.22%). Among the students in both institutions, 28.28% were overweight, making it the most common alteration (Figure 2).

The overall level of PA in the adolescents had a mean of 1.97 (95% CI 1.92 - 2.01) and was similar in both schools. When comparing each of the PAQ-A questions with school type, it was found that the level of activity in physical education classes PR= 0.67 (95% CI 0.53 - 0.85) and weekly intensity PR= 0.62 (95% CI 0.42 - 0.90) were higher in public school adolescents than in private school adolescents. However, students in the private school were more active before and after lunch PR= 1.94 (95% CI 1.58 - 2.39) than public school students (Table 2). Students engage in high-intensity PA on Thursdays and Fridays, while Saturdays and Sundays are the days when they have the least amount of PA. Public school students are more inactive on Mondays, Wednesdays, and Friday than private school students (Table 2).

**Table 2:** Physical Activity Questionnaire for Adolescents (PAQ-A) by type of school.

	Public institution	Private institution	PR (95%CI)	p^1^
* Active during physical education classes				
Low	70 (37.04)	85 (55.19)	Ref.	
High	119 (62.96)	69 (44.81)	0.67 (0.53 - 0.85)	<0.01
* Active before and after lunchtime				
Low	178 (94.18)	117 (75.97)	Ref.	
High	11 (5.82)	37 (24.03)	1.94 (1.58 - 2.39)	<0.01
* Active in the afternoon (14 - 18h)				
Low	153 (80.95)	120 (77.92)	Ref.	
High	36 (19.05)	34 (22.08)	1.10 (0.84 - 1.45)	0.15
Active on weekends				
Low	144 (76.19)	119 (77.27)	Ref.	
High	45 (23.81)	35 (22.73)	0.97 (0.73 - 1.28)	0.90
Weekly intensity				
Low	140 (74.07)	133 (86.36)	Ref.	
High	49 (25.93)	21 (13.64)	0.62 (0.42 - 0.90)	<0.01
High intensity per day				
Monday	28 (14.81)	52 (33.78)	1.68 (1.34 - 2.09)	<0.01
Tuesday	35 (18.52)	40 (25.97)	1.25 (0.97 - 1.62)	0.11
Wednesday	39 (20.63)	60 (38.96)	1.57 (1.26 - 1.97)	<0.01
Thursday	48 (25.40)	54 (35.07)	1.28 (1.00 - 1.62)	0.05
Friday	46 (24.34)	55 (35.71)	1.33 (1.05 - 1.68)	0.02
Saturday	39 (20.63)	31 (20.13)	0.98 (0.73 - 1.32)	1,0
Sunday	25 (13.22)	11 (7.14)	0.66 (0.39 - 1.09)	0.08
Physical activity during leisure time				
Mean (95%CI)	1.47 (1.40 - 1.55)	1.49 (1.42 - 1.55)	-	0.782
Physical activity level				
Mean (95%CI)	1.94 (1.88 - 2.00)	2.0 (1.94 - 2.06)	-	0.152

*During the last 7 days. PR: Prevalence Ratio; CI: Confidence interval. 1. Fisher exact test. 2. Test Student.

It was found that public school students were more inactive (adjusted PR= 1.40; 95% CI 1.03 - 1.92) compared to private school students. It was also observed that tenth and eleventh graders engage in less PA than ninth graders (Table 3).

**Table 3:** Factors associated with physical inactivity among adolescents.

	Physical inactivity <1.90	Physical activity >1.90	PR (95%CI)	p1	PR (95% CI) Adjusted1
Sex					
Female	92 (51.11)	88 (48.89)	Ref.		
Male	86 (52.76)	77 (47.24)	0.97 (0.79 - 1.19)	0.76	0.99 (0.80 - 1.24)
Type of school					
Public	109 (57.67)	80 (42.33)	Ref.		
Private	69 (44.81)	85 (55.19)	1.29 (1.04 - 1.61)	0.02	1.40 (1.03 - 1.92)
	Physical inactivity <1.90	Physical activity >1.90	PR (95%CI)	p1	PR (95% CI) Adjusted1
Grade level					
Ninth	63 (40.65)	92 (59.35)	1.0		1.0
Tenth	46 (58.23)	33 (41.77)	2.64 (1.09 - 1.87)	<0.01	1.43 (1.08 - 1.89)
Eleventh	69 (63.30)	40 (36.70)	3.65 (1.23 - 1.99)	<0.01	1.76 (1.38 - 2.26)
Socioeconomic stratification					
1	73 (57.94)	53 (42.06)	1.0		1.0
2	79 (50.00)	79 (50.00)	0.86 (0.70 - 1.07)	0.18	0.90 (0.70 - 1.13)
3	26 (44.07)	33 (55.93)	0.76 (0.54 - 1.03)	0.09	0.88 (0.55 - 1.35)
Excess weight					
No	113 (50.90)	109 (49.10)	Ref.		
Yes	52 (53.61)	45 (46.39)	0.95 (0.76 - 1.20)	0.65	1.05 (0.86 - 1.30)

*PR: Prevalence Ratio; CI: Confidence interval. 1. Log-binomial regression model*

## Discussion

To our knowledge, this is the first study in Colombia to compare nutritional status and PA levels with school type (public and private). The research showed that the level of PA was low in both schools, but students in the public school had higher levels of PI. In addition, a significant prevalence of excess weight was observed in the adolescents.

The study highlights a problem of public health importance as PI among adolescents in Colombia. Studies conducted in adolescents in countries such as Spain or the United States have reported PA levels (>2.6) ^24^^, ^^32^ higher than those found in this study. This situation is exacerbated when comparing by school type, as public-school students had a higher PI. This difference may be because private school adolescents engage in significantly more high-intensity PA three days a week (Monday, Wednesday, and Friday) than public school adolescents. This result was also found in a study of adolescents in Brazil, where more than 90% of students in a public school were physically inactive^23^.

In analyzing the differences in PA between public and private schools, it is important to examine the factors that might influence these differences. For example, it could be argued that there is unequal access to PA strategies and spaces in schools. It is plausible that private schools, generally associated with higher economic status, may have more resources to allocate to facilities and programs promoting PA among adolescents^16^^, ^^33^. Additionally, studies have shown the influence of socioeconomic factors, such as economic income, educational level, or social class of parents, on sedentary behaviors, including physical inactivity^22^.

It was also observed that tenth and eleventh graders did less PA than ninth graders. This finding is consistent with data from several studies showing that adolescents’ PA levels decrease with age, possibly due to screen-based leisure activities such as watching television and using mobile devices, video games, and others^33^^, ^^34^. The association between age and PA level has also been reported in studies conducted with adolescents in Greece^35^, the United States^36^, and Asian countries^37^. It has even been shown that the levels of PA are significantly reduced in older adults.

PI combined with a sedentary lifestyle is directly linked to an increase in body mass index, leading to overweight and obesity. It has medium- and long-term consequences, particularly an increased risk of chronic noncommunicable diseases^2^^, ^^3^^, ^^5^^, ^^6^. The increasing prevalence of overweight and obesity can have a negative impact on the quality of life of individuals and the well-being of society and represents a significant burden on countries’ health systems and economies^38^. Therefore, it is essential to promote physical activity among adolescents in various settings, both in and out of school, to reduce sedentary lifestyles and the risk of obesity in the future.

Given the high prevalence of nutritional excess and the low level of PA among adolescents, it is urgent to develop health promotion strategies, even with a differentiated approach according to the type of educational institution. Despite participation, adolescents do not achieve optimal levels of frequency and duration of PA, highlighting the need for educational programs and attractive interventions to promote PA. The results of this study have important implications for public health, particularly in school environments. We recommend the design and implementation of public policies that contribute to a healthy school environment, offer healthy food, and promote physical activity. Adolescence is an important stage in the development of healthy behaviors and habits^39^. The type of educational institution (private-public) could reflect the structural determinants of economic income and would generate social inequality in the level of physical activity of adolescents. This should attract attention at a political level.

The limitations of this study are worth noting. First, the lack of representativeness of the population at the national and regional levels (Huila, Colombia). The comparison between only two educational institutions limits the extent to which the study can provide a complete and accurate picture of the situation in a broader context. In addition, the limitation of the questionnaire used lies in its temporal scope, measuring the activity of the last seven days, excluding previous records; this could imply a loss of relevant data. Although validated in Spanish-speaking regions, this approach may have limitations in comprehensively measuring participants’ physical activity^32^. Additionally, the schools are from two municipalities with different social, demographic, and economic characteristics. This could have affected the comparison of the results, or there may have been variables that confounded the results.

## Conclusion

It is concluded that adolescent students have low levels of PA and that public school students are more inactive than private school students. The prevalence of excess weight was high in both educational institutions. It is important to conduct future studies with a larger sample size to investigate the social inequality of the level of physical activity by type of school. Qualitative studies that explore the particular circumstances of adolescents regarding healthy lifestyles, especially those with physical activity, are also recommended.
